# Endometriosis and Vesico-Sphincteral Disorders

**DOI:** 10.3389/fsurg.2015.00023

**Published:** 2015-06-22

**Authors:** Anis Fadhlaoui, Tessa Gillon, Issam Lebbi, Jean Bouquet de Jolinière, Anis Feki

**Affiliations:** ^1^Service de Gynécologie Obstétrique, HFR Fribourg – Hôpital Cantonal, Fribourg, Switzerland; ^2^Service de Gynécologie Obstetrique et de Médecine de la reproduction de l’Hôpital Aziza Othmana, Tunis, Tunisia; ^3^Faculté de Médecine de Tunis, Tunis, Tunisia; ^4^Obstetric Gynecology and Fertility Private Clinic, Dream Center, Tunis, Tunisia

**Keywords:** endometriosis, surgery, bladder, sphincter, deep endometriosis, urinary tract

## Abstract

**Objectives:**

The aim of this mini review is to determine the relationship between endometriosis and urinary tract symptoms and to investigate the consequences of surgical treatment of mild to severe endometriosis, especially deep lesions, on the vesico-sphincteral function (lower urinary tract function).

**Materials and methods:**

We performed a literature review by searching the MEDLINE database for articles published between 2000 and 2014, limiting the searches to the words: urinary tract, vesico-sphincteral, dysfunction, endometriosis, symptoms, and surgery.

**Results:**

The incidence of vesico-sphincteral symptoms in endometriosis varies from 3.4 up to 15.4%. The frequency of such symptoms seems to be under estimated because of a lack of specific questionnaire including urinary symptoms. Urodynamic evaluation could help to detect unsuspected abnormalities. It seems that endometriosis surgery (particularly deep infiltrating lesions) is a purveyor of de novo urinary dysfunction, with an incidence varying from 6.8 up to 17.5%. Nerve sparing processes such as neuro-navigators or neuro-stimulators seem to be promising techniques to avoid postoperative urinary tract dysfunction.

**Conclusion:**

A precise anamnesis and the use of specific validated questionnaires (IPSS and BFLUTS) improve the screening of vesico-sphincteral symptoms in case of endometriosis. No recommendation can be found in the literature about the place of urodynamic evaluation. Most publications lack of proof and therefore do not allow making recommendations about optimal treatment of endometriotic lesions to avoid urinary tract disorders.

## Introduction

Sampson gave the term “endometriosis” to a pathology primarily described in 1860 by Von Rokitansky. This pathology is a benign disease with high prevalence in women of reproductive age. Its etiology and pathogenesis are controversial but it is believed to involve multiple, genetic, environmental, immunological, angiogenic, endocrine, and even embryonic processes (1, 2). It is defined by the presence of endometrial and stromal glands out of the uterine cavity. It is estimated that 10–20% of women of childbearing age are affected (3), causing a variety of symptoms and diminishing their quality of life.

Vesico-sphincteral disorders such as voiding troubles are often masked by other symptoms of endometriosis such as pelvic pain (dysmenorrhea, dyspareunia, and chronic pelvic pain), menstrual cycle disturbances, and infertility.

The authors aim through this manuscript to update implications of endometriosis in vesico-sphincteral disorders.

## Background

Adenomyosis, previously called “internal endometriosis,” is distinguished from “external endometriosis,” actually simply called endometriosis. Adenomyosis is defined as the invasion of the myometrium by endometrial tissue (glands and stroma) (4). Endometriosis is defined as all other locations of endometrial tissue. Deep infiltrating endometriosis (DIE) is characterized by an infiltration of more than 5 mm of endometrial tissue into retroperitoneal space. Locations of DIE include the uterine torus, posterior fornix, uterosacral ligaments, rectum, vagina, and urinary tract (4).

The distribution of endometriotic lesions, regardless to their type (deep, superficial, or cyst-like), is heterogeneous, but the disease is most often pelvic-located (fallopian tubes, broad ligaments, ovaries, bladder and urethra, pelvic peritoneum including the Douglas’ pouch, utero-sacral ligaments, recto-vaginal septum, lower digestive system). Lesions are more likely situated in the posterior wall and on the left side of the pelvis (4). Ten percent of women with deep endometriosis show a gastrointestinal involvement. In those cases, involvement of the rectum and the recto-sigmoid portion occurs in 70–93% of the cases (4).

Involvement of the lower urinary tract is found in 0.2–2.5% of the cases (3, 5). Bladder and ureteral lesions occurred in, respectively, 25–85 and 15–75% of the cases, while renal and urethral involvement remains rare (5%) (3, 6).

Extra-pelvic endometriosis locations such as the perineum, laparotomy scars, trocar orifice, and *spinal cord* are reported in medical literature (3).

Two hypotheses about the etiology of endometriosis are still up to date and debated:

– The first one is the “*implantation theory*” (1), developed in 1927 by Sampson, suggesting that fragments of normal endometrium flow back into the peritoneal cavity during retrograde menstruations, passing through the tuba, then graft on different sites. Till nowadays, this hypothesis has found the largest approval. As a matter of fact, desquamated endometrial cells were found in the peritoneal cavity throughout the menstrual cycle. It seems rational to assume that these cells can implant on the peritoneum.
– The second one is the “*metaplasia theory*” (1), described in 1927 by Robert Meyer, which was based on suggestion that peritoneal tissue (Müllerian remnants) has a remaining metaplastic potential at adult age. This metaplastic phenomenon is more likely to occur under certain circumstances, such as estrogen influence and inflammatory phenomena.

Lower urinary tract endometriosis can be explained through several physio-pathological theories (5, 7), among them:

- *The Sampson’s migratory theory*, with implantation of endometrial cells in the vesico-uterine pouch.
- *The endometrial metaplasia* of Müllerian remnants located in the vesico-uterine pouch.
- *The invasive theory* corresponding to the extension of an anterior adenomyotic nodule.
- The iatrogenic bladder endometriosis starting from a cesarean scar.

It seems quite logical that a single theory can’t explain all clinical situations, but they are complementary.

## Physio-Pathological Explanations of Endometriosis-Related Urinary Tract Disorders

Pelvis innervation and voiding function are two complex systems that associate an autonomous and a voluntary nervous system for bladder emptying.

Bladder innervation is composed of:

– *A voiding control center* located in the brainstem and the sacral segment of the spinal cord.
– The peripheral nervous system divided into:

- *The cholinergic pelvic innervation* provided by the pelvic nerves with a stimulating role on the bladder and other smooth muscle cells.
- *The adrenergic innervation* provided by the hypogastric plexus with both relaxing and contracting function of the bladder and the ureter.
- *The somatic pudendal innervation* that commands the skeletal muscle cells (the external sphincter and the pelvic floor muscles).
- *The non-cholinergic non-adrenergic innervation* (NANC system) that has an afferent and efferent influence through neurotransmitters and neuromodulators.

Vesico-sphincteral disorders observed in endometriosis (day-time and night-time pollakiuria or urge incontinence, spontaneous and stress incontinence, urinary urgency, cystalgia, dysuria, urinary retention, cramps or pain during or at the end of urination, decreased bladder sensitivity, macroscopic hematuria, lower back pain…) imply an infiltration of the hypogastric plexus by endometriosis lesions, in combination with inflammatory phenomena. The involvement of the bladder, the ureter, or the urethra by endometriotic lesions directly explains some of these urinary symptoms (5, 8). According to Anaf et al., these events would be related to an overexpression of neural growth factors (9).

Medullary invasion by endometriotic nodules is an exceptional situation of the association between lower urinary tract disorders and endometriosis. Only seven cases (of medullary invasion) have been reported in literature with various neurological manifestations (hypoesthesia, weakness, failure of motor function), as well as constipation and urinary retention (10).

In case of pelvic endometriosis surgery, especially if involving the Douglas’ pouch, the rectum, the recto-sigmoïd junction, and the parameters, the postoperative bladder and sphincter disturbances are due to damages caused to the inferior hypogastric plexus that passes the utero-sacral ligaments laterally close to the rectum and the vaginal fornix (4, 9).

## Bladder and Sphincter Symptoms in Endometriosis

### Preoperative symptoms

The incidence of vesico-sphincteral symptoms in endometriosis seems low compared to other gynecological and digestive manifestations. It varies from 3.4 up to 15.4% (11) depending on the method used to highlight those symptoms. Ballester et al. (11) consider that this low incidence, especially in case of deep endometriosis, can be explained by the preponderance of gynecological and digestive symptoms. They also estimate that questionnaires used to diagnose endometriosis do not specifically include lower urinary symptoms items.

According to le Tohic et al. (5), in case of bladder endometriotic involvement, the catamenial rhythm of manifestations is an absolutely constant feature. Lower urinary symptoms were presented by cystalgia, dysuria, pollakiuria or urge incontinence, and macroscopic hematuria in, respectively, 58.3, 25, 16.6, and 12.5% of the cases.

In a study about preoperative evaluation of women with deep endometriosis, Boileau et al. reported an incidence of 65% of painful voiding and 4% dysuria, while the incidence of both gynecological and digestive symptoms was, respectively, 91 and 48% (4). In another study performed by Lapasse et al. (8) concerning 12 patients with deep endometriosis of the posterior pelvic wall, the anamnesis highlighted vesico-sphincteral symptoms among 50% of the patients (day-time and night-time pollakiuria, Urge incontinence, Bladder voiding by thurst, lower back pain, dysuria, full bladder cramps or pain, cramps or pain during or at end of urination, stress incontinence, decreased bladder sensitivity) (Table S1 in Supplementary Material).

In a comparative study between a group of patients carrying deep endometriosis and a control group, Ballester et al. (11), based on a validated questionnaire (IPSS: International Prostate Symptom Score) (12), showed a significantly higher incidence of voiding dysfunction in deep endometriosis group compared to control group (Table 1). Moreover, revealed urinary symptoms were dysuria, urge incontinence, spontaneous urinary incontinence, stress urinary incontinence, and cystalgia (when the bladder is full) in, respectively, 14.3, 28.7, 7.1, 15, and 30% of the cases.

**Table 1:** **Comparison of urinary symptoms using the IPSS Questionnaire (12)**.

IPSS	Endometriosis group		Control group		*p*
	Mean (DS)	95% CI	Mean (DS)	95% CI	
Symptoms of bladder filling	2.5 (3.1)	1.4–3.6	3.8 (3.7)	3.2–4.4	0.005
Symptoms of voiding	1.6 (1.5)	1.1–2.1	3.1 (3.1)	2.6–3.6	0.002
Total score	5.2 (5.3)	3.3–7.1	8.9 (7.8)	7.7–10.1	0.002

### Postoperative symptoms

Endometriosis surgery, in particular the one concerning deep infiltrating lesions with posterior involvement (such as recto-vaginal septum, cervical fornix, Douglas’ pouch, rectum, rectosigmoïd junction, and parameters), regardless of the operating technique (laparoscopic or open surgery), is a purveyor of *de novo* lower urinary (bladder and sphincter) dysfunction such as retention or dysuria. These disorders can be transient or persist in the longer term. Their incidence varies through studies from 6.8 to 17.5% (8). Vashisht et al. (13) reported that impaired bladder emptying occurred *de novo* in 14% of patients operated for a stage III or IV endometriosis according to the r-AFS classification of endometriosis. Dubernard and Volpi (14, 15) reported an incidence of *de novo* urinary dysfunction up to 17% of the cases after colorectal endometriosis. In a more recent prospective study by Daraï et al. (16), the authors evaluated the incidence of vesico-sphincteral dysfunction after deep endometriosis surgery, using two validated questionnaires (IPSS and BFLUTS: Bristol Female Lower Urinary Tract Symptoms); they showed that 29% of patients reported self catheterization for an average of 3 months; dysuria was observed in 29% of patients; urge incontinence in 11% of cases; spontaneous and stress incontinence in, respectively, 3 and 14% of cases. Another study (4) showed *de novo* voiding pain and dysuria were observed in, respectively, 6 and 18% of cases after deep endometriosis surgery. Dousset et al. (17) consider that the most common and specific complication following colorectal resections as part of the management of deep endometriosis is transient peripheral neurogenic bladder with an incidence of 16%.

## Urodynamic Evaluation

Deep endometriosis surgery, involving the uterosacral ligaments, the rectum, and recto-vaginal septum, induces (as described above) *de novo* vesico-sphincteral disorders caused by per operative nerve damage of the pelvic plexus. To distinguish preexisting disorders from those appeared *de novo*, some authors such as Lapasse et al. (8) propose to systematically carry out a preoperative urodynamic investigation in case of deep endometriosis (Table 2). To our knowledge, no other study about urodynamic evaluation in case of deep endometriosis has been published.

**Table 2:** **Preoperative urodynamic evaluation (8)**.

Normal urodynamic	2 cases (16.7%)
Significant post voiding residual	3 cases (25%)
Mean closure pressure	87, 8 ± 33.5 cm H_2_O
Urethral hypertonia	4 cases (30%)
Urethral instability	3 cases (25%)
Dysuria	3 cases (25%)
Hypersensitive bladder	2 cases (16.7%)
Sphincteral deficiency [closure pressure <110 − age]	2 cases (16.7%)
Bladder hypoesthesia	1 case (8.8%)
Reduced bladder capacity	1 case (8.8%)

The urinary flow study may reveal a multiphasic curve (related to urination with abdominal thrust), a decreased flow (<150 mL/s), and a significant post voiding residual (>10% of the urinated volume). Cystometric evaluation may show a hyper or hypo compliant bladder (hypo or hypersensitive bladder, flat curve, the first voiding-need may be delayed or absent), a detrusor hypocontractility with an increase in voiding duration, and/or a post-voiding residual. Urethral profilometry may be normal; however, a decreased urethral closure pressure is possible (in case of pudendal or vegetative nerve lesion) (18).

## Additional Investigations

### Imaging techniques

The radiological assessment in case of pelvic endometriosis (especially deep endometriosis) is based on ultrasound and MRI. In case of bladder or ureteral involvement, the trans-abdominal and/or trans-vaginal ultrasound can highlight echogenic nodular images at the vesico-uterine cul-de-sac infiltrating the detrusor. Given the multifocal nature of endometriotic lesions, the MRI remains the key investigation when viewing a hyper intense signal on T1 and persistent on T1 sequences with fat suppression reflecting the character of hemorrhagic lesions. For ureteral lesions, the T2-weighted MRI images provide identical images to that of intravenous urography cavities and bring the possibility to analyze the ureteral wall (19, 20).

### Endoscopic investigations

In the field of pelvic endometriosis, laparoscopy remains the gold standard. It allows the diagnosis (through macroscopic appearance and biopsy) and the treatment of the disease.

In case of bladder endometriosis, cystoscopy allows to visualize the lesions as blue submucosal bulging nodules. The interest of precise localization of those lesions related to the ureteral meatus is to evaluate the need of preoperative placement of an endo-prothesis (cystotomy vs ureterolysis) (20).

## Management

In most cases and regardless of the localization, the management of deep endometriosis lesions is surgical. The definitive diagnosis of endometriosis is histological, although a bundle of clinic-radiological findings guide toward the diagnosis (20). The surgical approach (conventional open surgery vs laparoscopy) depends on the team and surgeon’s expertise and skills. When comparing the delta values of urinary symptoms and quality of life using IPSS score, Ballester et al. (9) found no significant difference between laparoscopy and conventional open surgery. For voiding symptoms, ΔIPSS was 3.3 for laparoscopy and 1.3 for open surgery (*p*: 0.17), while for storage symptoms ΔIPSS was 1.2 for laparoscopy and −2.2 for open surgery (*p*: 0.16). Quality of life was comparable in both surgical routes (*p*: 0.45).

Different postoperative vesico-sphincteral manifestations (as mentioned above) as well as anatomical and physio-pathological data emphasize the need to develop a pelvic nerve-sparing surgery. Since the first publication of Possover et al. (21), several authors (11) have confirmed the feasibility of this surgical procedure (nerve-sparing surgery) with encouraging results. Indeed, Possover et al. (21) have developed the LANN technique, a technique of laparoscopic neuro-navigation to identify pelvic autonomic nerves. This reduces the incidence of postoperative bladder and sphincter disorders to <1%. Another method with encouraging results was proposed by the same team (22), by establishing a nerve stimulator on the hypogastric plexus to prevent bladder atony.

Regarding bladder lesions, the treatment of choice is partial cystectomy (full-thickness surgical excision of the bladder endometriosis lesion and its surrounding bladder wall), performed by classic laparoscopy or laparotomy (20, 23). Laparoscopic partial cystectomy offers the same results as laparotomy with several advantages such as less bleeding, shortened operative time, shortened hospital stay, quicker return to work, a major reduction in postoperative pain, and a major decreased risk of postoperative morbidity. To avoid recurrences and postoperative pain, the excision has to be as complete as possible. Concomitant cystoscopy can be useful for better defining the margins of the endometriotic lesion. Preventive catheterization of the ureters may be advisable, especially when the distance between the caudal border of the endometriotic lesion and the interureteric ridge is <2 cm (24). An accurate preoperative workup (anamnesis, clinical examination, and imaging information; cystoscopy) is essential for planning a correct laparoscopic approach. It is essential to ascertain the precise location of the vesical endometriotic nodule (the distance with the ureteral meatus and the lower endometriotic margins) (24).

Cystoscopy approach is not recommended and several arguments were against it. First, endometriotic bladder lesions develop inwards and complete resection involves accepting the risk of bladder perforation. Second, endometriosis being a disease of the peritoneal cavity, a complete assessment of the cavity is needed and cystoscopy does not allow this (20).

Ureteral lesions are treated by ureterolysis or resection/anastomosis based on intraoperative findings. When endometriotic lesions sheath the ureter, surgical procedure requires the placement of a stent before ureterolysis. Lateral involvement of the ureter usually requires resection/anastomosis (uretero-ureteral or uretero-vesical anastomosis) (20).

Robotic-assisted laparoscopy has the advantage of an improved level of freedom of instrumentation that allows more precise dissections, reduces nerve lesions (owing to 3D vision) and improves suturing techniques. However, the longer set-up time and greater expense limit its use for reference centers (24, 25).

Medical therapies for endometriosis may be used alone or as a complement to surgery. Prescribing medical treatment only implies certainty or at least a strong suspicion of the diagnosis. It is based on the sensitivity of the endometriotic tissue to hormones. The main treatments used are progestogens, local progestogens such as IUD with levonorgestrel, danazol, combined contraceptive pills, and GnRH analogs. Treatments success varies depending on the location of endometriosis lesions. Even when symptoms improve, it will tend to relapse when stopping treatment with a recurrence rate of 56% (24).

Aromatase inhibitors are approved adjuvants for the treatment of estrogen receptor-positive breast cancer. Molecular studies have revealed the presence of aromatase P450, the key enzyme in the biosynthesis of ovarian estradiol, inside the endometriotic tissue, indicating local synthesis of estradiol. Thereby, AIs represent an appealing medical option for the management of different aspects of this enigmatic disease, especially pelvic pain and infertility. Accordingly, this review aims to evaluate the potential role of AIs in the treatment of endometriosis-associated symptoms, mainly pain and infertility. Notably, several studies have demonstrated that the combination of AIs with conventional therapy as oral contraceptive pills, progestins, or gonadotropin-releasing hormone analogs can be used to control endometriosis-associated pain and pain recurrence in premenopausal women, particularly those with pain due to rectovaginal endometriosis refractory to other medical or surgical treatment. Some case reports have shown promising results in the treatment of postmenopausal endometriosis as first-line treatment, when surgery is contraindicated, or as second-line treatment in the case of postoperative recurrence (26).

In case of *de Novo* urinary disorders after surgery (urinary retention, dysuria, …), different drug treatments have been proposed, such as cholinergics or prokinetics (cisapride and bétanéchol), but their effectiveness has not been demonstrated yet. To date, standard treatment of urinary retention after surgery remains self-catheterization. Neuromodulation of the superior hypogastric plexus (inhibiting internal sphincter contraction and strengthening detrusor contraction) for treatment of refractory atonic bladder seems promising but should be confirmed by further studies. In terms of prevention, surgical nerve sparing techniques have been developed in order to minimize intraoperative injury of pelvic nerve plexus and reduce postoperative morbidity (18).

## Conclusion

It appears that bladder and sphincter disorders exist among patients with deep endometriosis regardless of its location, and thus before any surgery. The absence of previous symptoms couldn’t exclude endometriotic lesions. A precise anamnesis is fundamental to detect symptoms. The use of specific validated questionnaires, such as IPSS and BFLUTS, improve screening. Although it seems obvious that the implementation of urodynamic evaluation before surgery for deep endometriosis could help to detect unsuspected abnormalities, no recommendations can be found in recent literature. Localization of endometriosis lesions, such as in the posterior recto-vaginal septum, in Douglas’pouch, in the rectum and recto-sigmoïd junction, appears to be associated with a higher incidence of bladder and sphincter disorders. Surgery for deep endometriosis, in particular when extensive tissue resection is needed, is also responsible for *de novo* bladder and sphincter disorders. Processes such as the neuro-navigator and the neuro-stimulator placed laparoscopically seem promising techniques. That being said, most publications lack of proof and therefore do not allow extrapolating conclusions or making recommendations about optimal management of deep pelvic endometriosis related and its implication in vesico-sphinteral disorders.

## Conflict of Interest Statement

The authors declare that the research was conducted in the absence of any commercial or financial relationships that could be construed as a potential conflict of interest.

## Supplementary Material

The Supplementary Material for this article can be found online at http://journal.frontiersin.org/article/10.3389/fsurg.2015.00023
