# Supplementary material for: Endometriosis and Vesico-Sphincteral Disorders
Source: Front Surg. 2015 Jun 22;2:23. doi: 10.3389/fsurg.2015.00023 (PMC4476201; doi:10.3389/fsurg.2015.00023)
Supplement: Supplementary file 1 [file Table_1.DOCX]

Table S1: Lower urinary symptoms identified through questionnaire according to Lapasse et al (8)

| Symptoms | N | % |
| --- | --- | --- |
| Day time pollakiuria | 6 | 50 |
| Urge incontinence | 6 | 50 |
| Bladder voiding by thurst | 5 | 41,7 |
| Lower back pain | 4 | 33,3 |
| Dysuria | 3 | 25 |
| Night-time pollakiuria | 3 | 25 |
| Full bladder cramps or pain | 3 | 25 |
| Cramps or pain at end of urination | 3 | 25 |
| Stress incontinence | 2 | 16,7 |
| Decreased bladder sensitivity | 1 | 8,3 |
| Cramps or pain during urination | 0 | 0 |
